# Prediction of primary non-response to methotrexate therapy using demographic, clinical and psychosocial variables: results from the UK Rheumatoid Arthritis Medication Study (RAMS)

**DOI:** 10.1186/s13075-018-1645-5

**Published:** 2018-07-13

**Authors:** Jamie C. Sergeant, Kimme L. Hyrich, James Anderson, Kamilla Kopec-Harding, Holly F. Hope, Deborah P. M. Symmons, RAMS Co-Investigators, Adebajo Ade, Ahmed Khalid, Al-Ansari Atheer, Amarasena Roshan, Bukhari Marwan, Callan Margaret, G. Chelliah Easwaradhas, Chinoy Hector, Cooper Annie, Dasgupta Bhaskar, Davis Martin, Galloway James, Gough Andrew, Green Michael, Gullick Nicola, Hamilton Jennifer, Hassan Waji, Hider Samantha, Hyrich Kimme, Kamath Sanjeet, Knight Susan, Lane Suzanne, Lee Martin, Levy Sarah, Macphie Lizzy, Marguerie Christopher, Marshall Tarnya, Mathews Catherine, Mc Kenna Frank, Naz Sophia, Perry Mark, Pollard Louise, Quilty Brian, Robertson Lindsay, Roy Dipak, Sanders Paul, Saravanan Vadivelu, Scott David, Smith Gillian, Smith Richard, Symmons Deborah, Teh Lee-Suan, Viner Nick, Anne Barton, Suzanne M. M. Verstappen

**Affiliations:** 1 Centre for Biostatistics, Manchester Academic Health Science Centre, University of Manchester, Manchester, UK; 2 Arthritis Research UK Centre for Epidemiology, Centre for Musculoskeletal Research, Manchester Academic Health Science Centre, University of Manchester, Manchester, UK; 3 NIHR Manchester Biomedical Research Centre, Central Manchester University Hospitals NHS Foundation Trust, Manchester Academic Health Science Centre, Manchester, UK; 4 Arthritis Research UK Centre for Genetics and Genomics, Centre for Musculoskeletal Research, Manchester Academic Health Science Centre, University of Manchester, Manchester, UK

**Keywords:** Rheumatoid arthritis, Methotrexate, Response, Prediction model

## Abstract

**Background:**

Methotrexate (MTX) remains the disease-modifying anti-rheumatic drug of first choice in rheumatoid arthritis (RA) but response varies. Predicting non-response to MTX could enable earlier access to alternative or additional medications and control of disease progression. We aimed to identify baseline predictors of non-response to MTX and combine these into a prediction algorithm.

**Methods:**

This study included patients recruited to the Rheumatoid Arthritis Medication Study (RAMS), a UK multi-centre prospective observational study of patients with RA or undifferentiated polyarthritis, commencing MTX for the first time. Non-response to MTX at 6 months was defined as “no response” using the European League Against Rheumatism (EULAR) response criteria, discontinuation of MTX due to inefficacy or starting biologic therapy. The association of baseline demographic, clinical and psychosocial predictors with non-response was assessed using logistic regression. Predictive performance was assessed using the area under the receiver operating characteristic curve (AUC) and calibration plots.

**Results:**

Of 1050 patients, 449 (43%) were classified as non-responders. Independent multivariable predictors of MTX non-response (OR (95% CI)) were rheumatoid factor (RF) negativity (0.62 (0.45, 0.86) for RF positivity versus negativity), higher Health Assessment Questionnaire score (1.64 (1.25, 2.15)), higher tender joint count (1.06 (1.02, 1.10)), lower Disease Activity score in 28 joints (0.29 (0.23, 0.39)) and higher Hospital Anxiety and Depression Scale anxiety score (1.07 (1.03, 1.12)). The optimism-corrected AUC was 0.74.

**Conclusions:**

This is the first model for MTX non-response to be developed in a large contemporary study of patients commencing MTX in which demographic, clinical and psychosocial predictors were considered. Patient anxiety was a predictor of non-response and could be addressed at treatment commencement.

**Electronic supplementary material:**

The online version of this article (10.1186/s13075-018-1645-5) contains supplementary material, which is available to authorized users.

## Background

Methotrexate (MTX) is now the conventional synthetic disease-modifying antirheumatic drug (csDMARD) of first choice, either as monotherapy or combination therapy, for most patients with rheumatoid arthritis (RA) [1]. This is emphasised in a number of international and national guidelines [2–5]. However, response to MTX, although better than to most other csDMARDs, is not universal. In observational studies approximately 30% of patients discontinue MTX in the medium term - around half due to inefficacy and half due to adverse events [6, 7]. Patient-related factors such as female gender and current smoking are associated with MTX non-response [6, 7]. Disease related factors such as disease duration, disease activity, rheumatoid factor (RF) and anti-citrullinated protein antibody (ACPA) status are moderately predictive of inefficacy [6, 7]. Psychosocial factors may also be important but have received little attention to date [8]. Response to treatment may be influenced by the patient’s social background [9, 10], by their existing beliefs about their illness and the likely efficacy of the drug, and by whether they have actually taken the medication (adherence) [11]. Genetic or other biological factors may also influence drug response [6, 7].

Many previous studies have attempted to identify independent predictors of response to MTX, although frequently without assessing the ability to assign probabilities of response to individual patients [12–14]. Those prediction models that have been developed have used data from the restricted populations and rigid treatment regimens of clinical trials [15, 16]; have used only small numbers of participants (fewer than 100) from observational studies [17, 18]; or have analysed the outcome of treatment discontinuation rather than a broader assessment of patient condition [19]. The value of predictions from such models for “real world” patients with RA about to start MTX for the first time is uncertain. More likely to be of use is a model developed in an observational study including patients seen in routine clinical practice using readily available or easily measurable demographic, clinical and psychosocial factors. If such a model could identify those unlikely to respond to MTX prior to starting therapy, with sufficient accuracy to be clinically useful, it could enable earlier access to alternative medications such as biologic therapy and the avoidance of disease progression for some patients.

The objectives of this study were, in a large national multi-centre observational study of patients with RA or undifferentiated polyarthritis (UP) commencing MTX for the first time, to (1) describe the pattern of 6-month treatment response, (2) identify patient-specific, disease-specific and psychosocial predictors of primary non-response to MTX, (3) combine predictors of non-response in a model that could be used to assign probability of non-response at the individual patient level and (4) test the accuracy of the model.

## Methods

### Study design and study population

The Rheumatoid Arthritis Medication Study (RAMS) is a large national (UK) multi-centre (*n* = 38 centres) study. To be eligible for RAMS, patients had to (1) be aged 18 years or over, (2) have a physician diagnosis of RA or UP and (3) be about to start MTX for the first time, either as monotherapy or in combination with other csDMARDs, including oral steroids. Patients were not eligible if they had current or previous exposure to a biological DMARD (bDMARD). RAMS was approved by the Central Manchester NHS Research Ethics Committee (reference 08/H1008/25) and all patients provided written consent.

The decision to start MTX, the dosage and mode of administration, and whether to use MTX as monotherapy or in combination were made by the patient’s rheumatologist based on clinical need, local practice and national guidelines [3]. Patients were generally recruited following the drug education visit and prior to taking their first dose of MTX.

### Baseline assessments

Demographic and lifestyle data collected at baseline, and relevant to this analysis, included age, gender, height and weight to calculate body mass index (BMI), smoking status (current/former/never), current alcohol intake (units/fortnight) and current caffeinated tea and coffee consumption (cups/day). Socio-economic status was assigned using the Index of Multiple Deprivation (IMD) 2010 based on the patient’s postcode, where a higher IMD score represents a more deprived area [20].

Disease-specific data were collected from the patient by a research nurse and supplemented with information obtained from medical records, including symptom duration; 28 tender and swollen joint count; individual 1987 American College of Rheumatology (ACR) classification criteria for RA [21]; previous csDMARD history; current oral steroid use; intramuscular or intra-articular steroid injections in the past week; current use of non-steroidal anti-inflammatory drugs (NSAIDs); duration of morning stiffness; and serum creatinine. Self-reported comorbidities were selected from a list of predefined conditions (high blood pressure, angina, heart attack, transient ischaemic attack, stroke, epilepsy, asthma, chronic bronchitis/emphysema, bronchiectasis, peptic ulcer disease, liver disease, renal disease, tuberculosis, diabetes mellitus, hyperthyroidism, depression and cancer).

Patients also completed a questionnaire, including pain, fatigue and general well-being visual analogue scales (VAS) (0–100 mm, with 100 mm the worst score); the British version of the Health Assessment Questionnaire (HAQ) (score range 0–3) [22]; the Hospital Anxiety and Depression Scale (HADS) (score ranges 0–21) [23] and the Beliefs about Medicines Questionnaire (BMQ) (score ranges 5–25) [24], with higher values of the HAQ, HADS and BMQ representing reduced physical function, greater indication of anxiety or depression and stronger beliefs about medication necessity or concerns, respectively. The brief Illness Perception Questionnaire (IPQ-brief) [25] was used to categorise patients’ illness representations as positive or negative [26].

Blood samples were taken at baseline and sent to the UK Biobank, Stockport, UK for the measurement of C-reactive protein (CRP) (Beckman Coulter AU5400, CRP assay OSR6147; mg/l) and RF (Beckman Coulter AU5400, RF latex assay OSR61105; IU/ml). RF values in excess of 14 IU/ml were taken to indicate RF positivity. If blood samples were not available to measure CRP, recorded CRP values from medical notes were used. The DAS28 was calculated using the CRP, 28-joint counts and VAS for general well-being [27].

### Follow-up assessments

Patients were followed up at 3 and 6 months. Changes in DMARD therapy, including MTX, were recorded and the DAS28-CRP was measured at each visit. If MTX therapy had been stopped, the reason for stopping and whether treatment would be restarted were also recorded.

### Outcome: European League Against Rheumatism (EULAR) non-response

Non-response to treatment at 6 months was defined as “no response” using the EULAR response criteria [28], i.e. Disease Activity Score in 28 joints (DAS28) improvement ≤ 0.6, or DAS28 improvement > 0.6 but ≤ 1.2 and 6-month DAS28 > 5.1. In addition, patients who had discontinued MTX by 6 months, i.e. had stopped MTX and did not plan to restart, due to inefficacy were classified as non-responders, as were patients who commenced bDMARD treatment by 6 months. “Moderate” or “good” responders by the EULAR criteria were considered responders, as were patients who discontinued MTX by 6 months due to remission.

### Participant selection

**Fig. 1:**
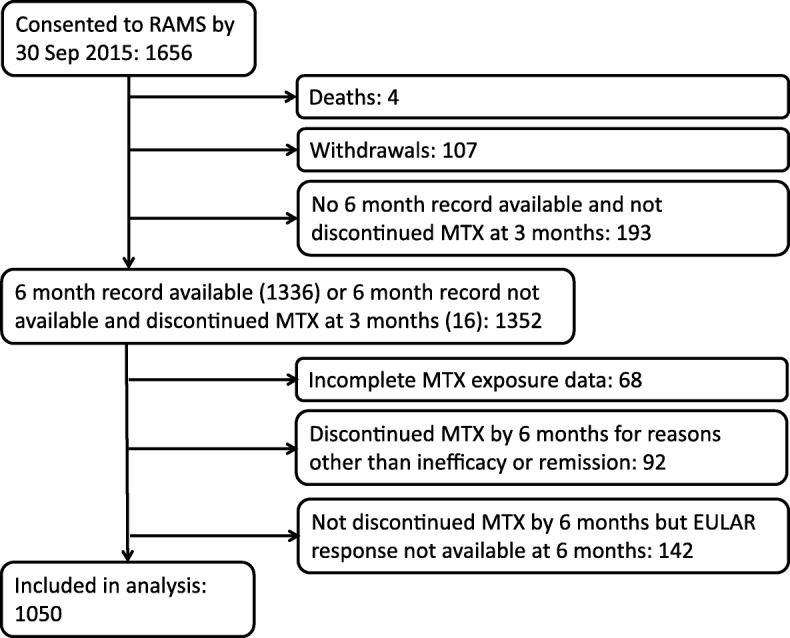
Flow diagram of participant inclusion. RAMS Rheumatoid Arthritis Medication Study; MTX, methotrexate; EULAR, European League Against Rheumatism

To allow for sufficient follow-up time, the current analysis included RAMS participants recruited by 30 September 2015. Patients without a 6-month follow-up record were excluded, unless they had discontinued MTX by their 3-month follow up and so could be classified as non-responders. Also excluded were patients with unknown MTX exposure status at 6 months, those who had discontinued MTX by 6 months for reasons other than inefficacy or remission (e.g. adverse events), and those who had not discontinued MTX by 6 months but for whom the 6-month EULAR response was unavailable (see Fig. 1). If MTX or restart status at 6 months was unknown, 3-month records were checked for evidence of having discontinued at this time point before excluding a patient.

### Statistical analysis

All variables were assessed for their univariable and multivariable association with non-response to MTX at 6 months using logistic regression. Backward selection was used to successively remove non-significant terms (*p* ≥ 0.05) from a full multivariable model containing all variables. Forwards selection was also used to successively add significant predictors (*p* < 0.05) into an empty model to validate the list of predictors derived by backwards selection. If the backwards and forwards selection processes delivered different sets of predictors, backwards selection was applied again on the pooled set of predictors derived from both approaches to produce a final model. The ability of the final model to discriminate between responders and non-responders was assessed using the area under the receiver operating characteristic curve (AUC). Agreement between predicted probabilities and observed outcomes was assessed using a calibration plot of the observed proportions of non-responders in each decile of predicted probability of non-response plotted against the mean predicted probabilities for the deciles. As performance was assessed using the same data used to build the model, an estimate of the optimism in the AUC value was produced using 200 bootstrapped datasets. The modelling procedure was followed afresh in each bootstrap dataset and the estimate of optimism was calculated as the average difference between the AUC achieved by a model in its own bootstrap dataset and the AUC achieved by that model in the original dataset [29]. How the model might perform in clinical practice was explored by calculating the sensitivity, specificity, positive predictive value (PPV) and negative predictive value (NPV) when using different cut-offs of predicted probabilities of non-response as thresholds for classifying individuals as having a high risk of non-response.

Rates of missing data were calculated for all potential predictor variables and an analysis using multiple imputation with chained equations to impute missing values in all candidate predictor variables in 50 imputed datasets was performed. All analyses were performed in Stata 13.1 [30].

## Results

Of 1656 patients recruited by 30 September 2015, 1050 were included in the current analysis (Fig. 1): 707 (67%) female, median age 59 (IQR 49–68) years and median symptom duration 9 (IQR 4–28) months (Table 1). Of the patients, 66% (584/889) were RF positive and 82% (787/962) satisfied the 1987 ACR criteria for RA at baseline; 77% of patients were starting MTX as their first csDMARD; 18% were currently on another csDMARD; and 4% had prior but not current exposure to one or more csDMARDs (Table 1): 41% were taking oral corticosteroids and/or had recently (within the last week) received an intramuscular corticosteroid injection, and 3% of patients had received an intra-articular steroid injection in the previous week (Table 1). Almost all participants (1003/1005) were starting folic acid at baseline. Starting doses of MTX were recorded at baseline for 1042 (99%) participants and ranged from 2.5 to 25 mg/week, with plans to incrementally increase the dose in 43% (422/984) of cases; 98% (963/978) of participants were starting orally administered MTX.

**Table 1:** Baseline characteristics of the whole cohort and divided by responder status

Characteristic	Data availability	All patients (*n* = 1050)	Responders (*n* = 601)	Non-responders (*n* = 449)
Demographic and lifestyle factors				
Female sex	1050 (100)	707 (67)	398 (66)	309 (69)
Age (years)	1050 (100)	59 (49, 68)	60 (49, 68)	58 (48, 67)
BMI (kg/m^2^)	959 (91)	27.5 (24.2, 31.6)	27.4 (24.1, 31.1)	27.9 (24.3, 33.0)
Smoking: never	1042 (99)	420 (40)	260 (44)	160 (36)
Smoking: former		404 (39)	232 (39)	172 (39)
Smoking: current		218 (21)	104 (17)	114 (26)
Alcohol consumption: current	1030 (98)	710 (69)	401 (68)	309 (71)
Alcohol consumption: (units/fortnight)	1009 (96)	2 (0, 10)	2 (0, 10)	2 (0, 12)
Coffee/tea consumption: (cups/day)	815 (78)	4 (3, 6)	4 (2, 6)	4 (3, 6)
IMD score	990 (94)	13.9 (8.9, 24.5)	13.7 (8.5, 23.7)	14.8 (9.5, 27.2)
Disease-specific factors				
Symptom duration (months)	1042 (99)	9 (4, 28)	8 (4, 24)	10 (4, 33)
RF positive	889 (85)	584 (66)	354 (70)	230 (60)
Satisfied the 1987 ACR criteria	962 (92)	787 (82)	470 (85)	317 (77)
HAQ score	989 (94)	1.1 (0.5, 1.6)	1.1 (0.5, 1.8)	1.0 (0.4, 1.5)
Co-morbidities: 0	1050 (100)	392 (37)	240 (40)	152 (34)
Co-morbidities: 1		345 (33)	189 (31)	156 (35)
Co-morbidities: 2+		313 (30)	172 (29)	141 (31)
Creatinine (mg/dl)	970 (92)	67 (59, 77)	66 (58, 76)	67 (60, 79)
Disease activity				
Morning stiffness (minutes)	1000 (95)	60 (15, 120)	60 (20, 120)	60 (10, 90)
TJC28	1050 (100)	6 (2, 13)	8 (4, 15)	4 (1, 10)
SJC28	1050 (100)	5 (2, 10)	6 (3, 12)	3 (1, 7)
CRP (mg/l)	1050 (100)	5.6 (2.0, 16.2)	8.2 (2.8, 21.4)	3.8 (1.5, 9.9)
Patient VAS (mm)	1050 (100)	41 (23, 60)	47 (26, 66)	32 (19, 50)
DAS28-CRP	1050 (100)	4.3 (3.3, 5.3)	4.7 (3.9, 5.7)	3.7 (2.8, 4.6)
DAS28-CRP ≤ 3.2	1050 (100)	226 (22)	64 (11)	162 (36)
DAS28-CRP ≤ 2.6	1050 (100)	102 (10)	12 (2)	90 (20)
Pain VAS (mm)	971 (92)	50 (28, 71)	53 (30, 73)	46 (25, 68)
Fatigue VAS (mm)	974 (93)	54 (27, 73)	54 (28, 74)	53 (27, 72)
Medication				
Oral steroids: current	1048 (100)	210 (20)	119 (20)	91 (20)
Intramuscular steroids: recent	1028 (98)	234 (23)	137 (23)	97 (22)
Intra-articular steroids: recent	882 (84)	27 (3)	14 (3)	13 (3)
Oral or intramuscular steroids: current/recent	1031 (98)	423 (41)	244 (41)	179 (40)
NSAIDs: current	942 (90)	520 (55)	296 (55)	224 (55)
csDMARDs: current	1050 (100)	193 (18)	100 (17)	93 (21)
csDMARDs: ever	1050 (100)	238 (23)	120 (20)	118 (26)
MTX starting dose (mg/week)	1042 (99)	10 (10, 15)	10 (10, 15)	10 (10, 15)
Psychosocial factors				
HADS anxiety	986 (94)	6 (3, 9)	6 (3, 9)	6 (3, 10)
HADS depression	987 (94)	6 (3, 9)	6 (3, 9)	5 (2, 8)
BMQ medication necessity	942 (90)	19 (17, 22)	20 (17, 23)	19 (17, 22)
BMQ medication concerns	949 (90)	15 (13, 18)	15 (12, 17)	16 (13, 18)
BMQ necessity-concerns	923 (88)	4 (1, 8)	5 (1, 8)	4 (1, 8)
IPQ negative illness representation	967 (92)	560 (58)	330 (59)	230 (56)

Values are frequency (%) or median (IQR)

*Abbreviations: BMI* body mass index, *IMD* Index of Multiple Deprivation, *RF* rheumatoid factor, *HAQ* Health Assessment Questionnaire, *TJC28* tender 28-joint count, *SJC28* swollen 28-joint count, *CRP* C-reactive protein, *VAS* visual analogue scale, *DAS28* Disease Activity Score based on the 28-joint count, *NSAIDs* non-steroidal anti-inflammatory drugs, *csDMARDS* conventional synthetic disease-modifying anti-rheumatic drugs, *HADS* Hospital Anxiety and Depression Scale, *BMQ* Beliefs about Medicines Questionnaire, *IPQ* Illness Perception Questionnaire

### Outcome: non-response

At 6 months 449/1050 patients (43%) were classified as non-responders. Table 1 gives baseline characteristics stratified by response status at 6 months. In the univariable analysis, significant predictors of MTX non-response (OR (95% CI)) included higher BMI (1.02 (1.00, 1.05) per kg/m^2^), current smoking (1.78 (1.28, 2.48) compared to never smoking), longer symptom duration (1.00 (1.00, 1.00) per month); not being RF positive (0.66 (0.50, 0.88) for RF positive compared to not); not satisfying the 1987 ACR criteria (0.59 (0.43, 0.83) for satisfying the 1987 ACR criteria compared to not); lower HAQ score (0.76 (0.64, 0.90) per unit increase in HAQ) and lower DAS28 (0.54 (0.48, 0.60) per unit increase in DAS28) (Table 2). In the multivariable model, not being RF positive (0.62 (0.45, 0.86) for RF positive compared to not), higher HAQ score (1.64 (1.25, 2.15) per unit increase in HAQ), higher tender joint count (1.06 (1.02, 1.10) per additional tender joint), lower DAS28 score (0.29 (0.23, 0.39) per unit increase in DAS28) and higher HADS anxiety score (1.07 (1.03, 1.12) per unit increase in HADS anxiety) were independent predictors of MTX non-response (Table 2). The results using 50 multiple imputation datasets to account for missing values were almost identical (results not shown).

**Table 2:** Univariable and multivariable analysis of predictors of MTX non-response in all subjects and excluding those in remission at baseline

Characteristic	All subjects				Excluding those in remission			
	Univariable		Stepwise multivariable *n* = 833, AUC = 0.77 (95% CI (0.73, 0.80))		Univariable		Stepwise multivariable *n* = 700, AUC = 0.72 (95% CI (0.68, 0.76))	
	OR (95% CI)	*p*	OR (95% CI)	*p*	OR (95% CI)	*p*	OR (95% CI)	*p*
Demographic and lifestyle factors								
Female sex	1.13 (0.87, 1.46)	0.37			1.20 (0.90, 1.59)	0.22		
Age (years)	0.99 (0.98, 1.00)	0.06			0.99 (0.98, 1.00)	0.04		
BMI (kg/m^2^)	1.02 (1.00, 1.05)	0.04			1.04 (1.02, 1.06)	< 0.01	1.05 (1.02, 1.08)	< 0.01
Smoking: never	ref	ref			ref	ref		
Smoking: former	1.20 (0.91, 1.59)	0.19			1.23 (0.91, 1.65)	0.18		
Smoking: current	1.78 (1.28, 2.48)	< 0.01			1.74 (1.22, 2.48)	< 0.01		
Alcohol consumption: current	1.14 (0.87, 1.49)	0.34			1.13 (0.85, 1.51)	0.40		
Alcohol consumption: (units/fortnight)	1.01 (1.00, 1.02)	0.08			1.01 (1.00, 1.02)	0.21		
Coffee/tea consumption: (cups/day)	1.03 (0.98, 1.09)	0.20			1.04 (0.99, 1.10)	0.12		
IMD score (units)	1.01 (1.00, 1.02)	0.02			1.01 (1.00, 1.02)	0.05		
Disease-specific factors								
Symptom duration (months)	1.00 (1.00, 1.00)	< 0.01			1.00 (1.00, 1.01)	< 0.01		
RF positive	0.66 (0.50, 0.88)	< 0.01	0.62 (0.45, 0.86)	< 0.01	0.57 (0.42, 0.76)	< 0.01	0.52 (0.37, 0.73)	< 0.01
Satisfied the 1987 ACR criteria	0.59 (0.43, 0.83)	< 0.01			0.66 (0.46, 0.94)	0.02		
HAQ score (units)	0.76 (0.64, 0.90)	< 0.01	1.64 (1.25, 2.15)	< 0.01	0.92 (0.77, 1.11)	0.39		
Co-morbidities: 0	ref	ref			ref	ref		
Co-morbidities: 1	1.30 (0.97, 1.75)	0.08			1.45 (1.06, 2.00)	0.02		
Co-morbidities: 2+	1.29 (0.96, 1.75)	0.09			1.50 (1.08, 2.07)	0.01		
Creatinine (mg/dl)	1.01 (1.00, 1.01)	0.10			1.01 (1.00, 1.01)	0.18		
Disease activity								
Morning stiffness (minutes)	1.00 (1.00, 1.00)	0.01			1.00 (1.00, 1.00)	0.18		
TJC28 (joints)	0.94 (0.92, 0.96)	< 0.01	1.06 (1.02, 1.10)	< 0.01	0.96 (0.95, 0.98)	< 0.01		
SJC28 (joints)	0.92 (0.90, 0.94)	< 0.01			0.94 (0.92, 0.96)	< 0.01		
CRP (mg/l)	0.98 (0.97, 0.99)	< 0.01			0.99 (0.98, 0.99)	< 0.01		
Patient VAS (mm)	0.98 (0.97, 0.99)	< 0.01			0.99 (0.98, 0.99)	< 0.01		
DAS28-CRP (units)	0.54 (0.48, 0.60)	< 0.01	0.29 (0.23, 0.39)	< 0.01	0.63 (0.55, 0.71)	< 0.01	0.49 (0.41, 0.58)	< 0.01
Pain VAS (mm)	0.99 (0.99, 1.00)	< 0.01			1.00 (0.99, 1.00)	0.26		
Fatigue VAS (mm)	1.00 (0.99, 1.00)	0.29			1.00 (1.00, 1.01)	0.21		
Medication								
Oral or intramuscular steroids: current/recent	0.96 (0.75, 1.24)	0.76			0.97 (0.74, 1.27)	0.82		
NSAIDs: current	1.02 (0.78, 1.32)	0.90			1.08 (0.82, 1.43)	0.58		
csDMARDs: current	1.31 (0.96, 1.79)	0.09			1.37 (0.99, 1.91)	0.06		
csDMARDs: ever	1.43 (1.07, 1.91)	0.02			1.50 (1.11, 2.04)	0.01		
MTX starting dose (mg/week)	0.97 (0.94, 1.02)	0.22			0.98 (0.94, 1.02)	0.30		
Psychosocial factors								
HADS Anxiety (units)	1.02 (0.99, 1.05)	0.21	1.07 (1.03, 1.12)	< 0.01	1.04 (1.01, 1.07)	0.01	1.11 (1.07, 1.15)	< 0.01
HADS Depression (units)	0.98 (0.95, 1.01)	0.19			1.01 (0.98, 1.05)	0.57		
BMQ medication necessity (units)	0.98 (0.94, 1.01)	0.23			0.99 (0.95, 1.03)	0.50		
BMQ medication concerns (units)	1.03 (0.99, 1.06)	0.14			1.03 (0.99, 1.07)	0.14		
BMQ necessity-concerns (units)	0.98 (0.95, 1.00)	0.08			0.98 (0.95, 1.01)	0.16		
IPQ negative illness representation	0.88 (0.68, 1.14)	0.33			1.14 (0.86, 1.51)	0.37		

*Abbreviations: BMI* body mass index, *IMD* Index of Multiple Deprivation, *RF* rheumatoid factor, *HAQ* Health Assessment Questionnaire, *TJC28* tender 28-joint count, *SJC28* swollen 28-joint count, *CRP* C-reactive protein, *VAS* visual analogue scale, *DAS28* Disease Activity Score based on the 28-joint count, *NSAIDs* non-steroidal anti-inflammatory drugs, *csDMARDS* conventional synthetic disease-modifying anti-rheumatic drugs, *HADS* Hospital Anxiety and Depression Scale, *BMQ* Beliefs about Medicines Questionnaire, *IPQ* Illness Perception Questionnaire

### Sensitivity analysis

The most surprising result was the relationship between a lower DAS28 score and non-response. This may be an inevitable consequence of the method of calculating non-response. In order to be classified as a responder to MTX by achieving a moderate or good EULAR response, it is necessary for a patient’s DAS28 score to fall by at least 0.6. If patients start with a relatively low DAS28 score they have less potential for achieving such a response. Indeed, since the DAS28-CRP(4) formula includes a constant of 0.96, a DAS28 of 1.56 is the minimum that can fall by 0.6 or more and be classified as response. At baseline, 226 (22%) patients had low disease activity (LDA) (DAS28-CRP ≤ 3.2), including 102 (10%) patients in remission (DAS28-CRP ≤ 2.6). Of these, 72% (162/226) and 88% (90/102), respectively, were non-responders, compared to 43% of all patients (Table 1). 40% (88/219) of those with LDA and 38% (38/99) of those in remission were on oral corticosteroids at baseline or had received an intramuscular corticosteroid injection in the past week, similar to the 41% of the whole cohort.

To further explore the role of baseline DAS28, we conducted two sensitivity analyses, first excluding patients in remission and second excluding patients with LDA but otherwise following the modelling procedure described above. The multivariable models excluding those in remission and those with LDA contained the same predictors as the main model except for the addition of BMI and the removal of HAQ score and TJC28 (Table 2 and Additional file 1: Table S1). Hence the predictors common to all three multivariable models were RF status, DAS28-CRP and HADS anxiety score.

Additionally, a model was developed to predict failure to achieve LDA (i.e. DAS28-CRP > 3.2) at 6 months (not requiring a minimum improvement in DAS28). Higher BMI, higher HAQ score, higher TJC28 and higher HADS anxiety score, but not lower baseline DAS28, were predictive of failing to achieve LDA at 6 months (Additional file 1: Table S2).

### Model assessment

**Fig. 2:**
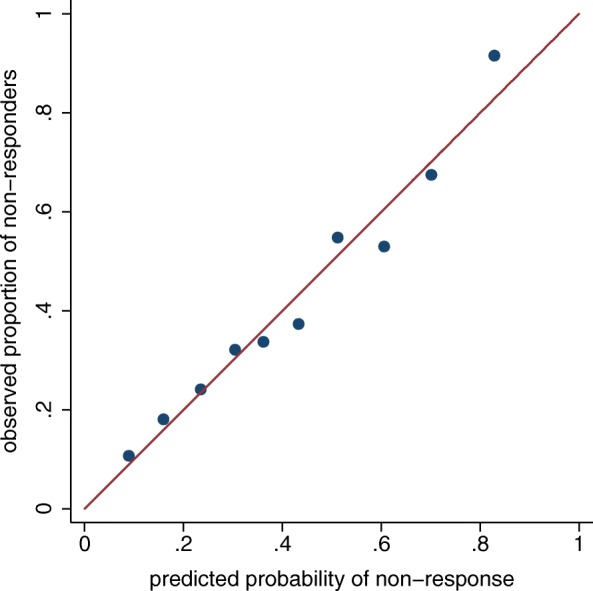
Calibration plot for multivariable prediction model for non-response to methotrexate

The model predicting non-response in all patients had an AUC of 0.77 (95% CI (0.73, 0.80)) (Table 2), reduced to 0.74 when correcting for optimism. Excluding those in remission or with LDA reduced the AUC to 0.72 (0.68, 0.76) (Table 2) and 0.71 (0.66, 0.75) (Additional file 1: Table S1), respectively. Calibration plots in Fig. 2 and Additional file 1: Figures S1 and S2 indicate that the models have similar properties across the deciles of predicted probabilities. For the outcome of “failure to achieve LDA at 6 months”, the AUC was 0.73 (0.70, 0.77) (Additional file 1: Table S2) and the calibration plot is shown in Additional file 1: Figure S3.

Table 3 shows the sensitivity, specificity, PPV and NPV when predicted probability cut-offs ranging from 0.5 to 0.9 are used as thresholds for classifying individuals as being at high risk of non-response. Using a cut-off of 0.8 to indicate high risk, 49 patients (6%) would be predicted to be at high risk of non-response and 98% of these would indeed fail to respond (PPV); 61% of those predicted to respond would do so (NPV). On average the model would need to be applied to 17 patients to identify one individual at high risk of non-response (“number needed to test”).

**Table 3:** Sensitivity (Sen), specificity (Spec), positive predictive value (PPV) and negative predictive value (NPV) for a range of probability cut-offs for classifying those at high risk of non-response (main model)

Cut-off	Predicted non-responders	Sen	Spec	PPV	NPV
0.5	303 (36%)	59%	80%	68%	72%
0.6	210 (25%)	45%	89%	75%	69%
0.7	123 (15%)	30%	96%	86%	65%
0.8	49 (6%)	14%	100%	98%	61%
0.9	13 (2%)	4%	100%	100%	59%

## Discussion

In this large observational study investigating response to MTX among patients with RA in the current era, 43% of patients were classified as non-responders by 6 months after starting treatment, with those discontinuing MTX due to adverse events excluded from the analysis. Baseline predictors of non-response in a multivariable logistic regression model were RF negativity, higher HAQ score, higher tender joint count, higher HADS anxiety score and lower disease activity. The AUC was 0.77 (0.74 optimism-corrected). The AUC was lower in models that excluded either all those in remission or all those with LDA at baseline when attempting to address the fact that it was harder for those with lower baseline DAS28 scores to achieve the definition of response. All models included RF negativity, lower baseline DAS28-CRP and higher HADS anxiety score as predictors of non-response. This is the first study to explore potential psychological factors in prediction of individual MTX non-response and all models retained HADS anxiety score as an independent predictor. Hence this psychological predictor added significant additional predictive information once the clinical predictors in the multivariable model had been accounted for. While the design of the current study does not allow us to examine the mechanism by which anxiety is associated with non-response, and this relationship requires further research, anxiety could be considered a modifiable risk factor, suggesting that the shared decision-making between patient and rheumatologist [2] should be mindful of anxiety issues, and that patient education prior to starting MTX should address anxiety. Although the literature on the association between patient anxiety and response to treatment in RA is limited, a recent study [31] did report that depression and anxiety (without differentiating between the two) may reduce likelihood of remission in those treated with MTX or tumour necrosis factor inhibitors.

While previous attempts have been made to develop models to predict response to MTX [15–19], this is the first model to be developed using a large cohort of patients with RA starting MTX for the first time and recruited from routine clinical care with few exclusions, i.e. representative of the setting in which such a model might be applied. That is, our model is designed to be applied in the real-world population of those about to commence MTX, which includes individuals with disease activity lower than might be expected. Final sets of predictor variables vary between published models, with only some measure of patient condition at baseline, be it TJC [15], DAS [16, 17] or HAQ [19], common to most studies. While lower baseline DAS28 was found to be associated with non-response to MTX when defined primarily using the EULAR response criteria, higher baseline DAS28 was associated with failure to achieve LDA (although significant only univariably, with higher TJC being retained in the multivariable model), which matches findings elsewhere [16]. This is a reminder that models are outcome-specific and that the clinical relevance of outcomes should be considered.

If a predicted probability of 0.9 or above from the model was used to identify patients at high risk of non-response, 100% of those meeting this criterion would go on to be non-responders. However, only 4% of non-responders would be identified in this way. Reducing the cut-off to 0.8 would identify 14% of non-responders, but at the expense of 2% of those labelled as high risk actually being patients who would respond to MTX. The trade-off between the delay in accessing alternative medications for those who will not respond to MTX but are predicted to do so, and the over-treatment with alternative medications of those who would respond to MTX but are predicted not to, is unlikely to be an equally weighted one. Deciding where to draw an appropriate threshold for a label of high risk for clinical practice requires consideration of the treatment options available in a particular setting, their benefits and risks for individual patients, and their health economic implications. Of course, even with perfect prediction of non-response to MTX therapy, there is no guarantee that a better response would be achieved with alternative treatments. Truly informed decision-making by clinicians and their patients would require personalised predictions of patient outcomes for a range of treatment options, a scenario which is still some way off.

The strengths of this analysis include the large sample size and the fact that it reflects use of MTX according to current guidelines and so supersedes earlier work [12–19]. The definition of non-response at 6 months embraced those who remained on the drug but had not exhibited enough improvement to be classified as moderate or good EULAR responders and also those who had discontinued the drug due to inefficacy or started a bDMARD. Other strengths were the inclusion of potential psychological predictors and using multiple imputation to provide reassurance of the robustness of the results to missing predictor data.

This study also has some limitations. The non-response rate was high. This may, in part, be due to suboptimal dosing or route of administration. Although the study did not dictate the treatment protocol, all patients should have been managed according to national guidelines [3] in which escalation of MTX is permitted and combination therapy is encouraged. We do not know the reasons for any deviations from treatment guidelines, but this prediction model may help guide clinicians as to which patients are less likely to respond as they are currently practicing and encourage them to consider treating more intensively. As we have attempted to predict non-response using only information available before the commencement of MTX, we have not considered the relationship between time-varying MTX dose and non-response. Titration is highly influenced by starting dose and patient response to treatment. The goal of the current work is to try and predict response prior to the start of MTX and, although maximum MTX dose and rate of titration may also be associated with response, that information would not be available pre-treatment. Further research is required to specifically investigate how rate and characteristics of MTX titration may also influence response, taking early response and adverse events into account. It seems likely that the observed association between lower DAS28 at baseline and subsequent non-response is explained by less scope for improvement (the key component of response) for those with lower disease activity. Since patients were recruited from routine clinical care, a high proportion of patients (41%) received oral or intramuscular steroids between the decision to prescribe MTX and the baseline assessment. We therefore performed sensitivity analysis, stratifying by steroid use, and the results were very similar (results not shown). The aim of this study was to use demographic, clinical and psychosocial variables that are readily available or easily measurable, to predict non-response. In future we aim to add genetic and metabolic predictors with the hope of improving the accuracy and clinical applicability of the model. Finally, these models were only validated internally. RAMS continues to recruit new patients so there will be an opportunity for further internal validation (“temporal validation”) in the future. However, external validation in an independent dataset, which includes information on the relevant predictor variables that would be needed before the models could be considered for clinical use.

It might be reasonable to consider by-passing MTX therapy altogether in patients predicted to be very unlikely to respond. This model assigns ≥ 90% probability of non-response to only a tiny proportion (2%) of patients. Would it be reasonable to accept a lower probability of non-response as a guide to MTX prescription - or a guide to starting combination therapy? This depends to some extent on the alternative forms of treatment and their efficacy in individuals predicted not to respond to MTX, and their cost. In the current situation it seems reasonable to continue to prescribe MTX for most patients in whom it is not contra-indicated but with a low threshold to move on to stronger combination or biological therapy if there is non-response at 6 months.

## Conclusions

We have developed a model to predict non-response to MTX using data from a large contemporary observational study of patients with RA and UP commencing MTX for the first time. This is the first such model to consider patient-specific, disease-specific and psychosocial predictors. Using a high predicted probability to classify patients as at high risk of non-response would identify a small proportion of such individuals with perfect specificity. Patient anxiety was a multivariable predictor of non-response to MTX, a relationship that requires further research and which could be addressed prior to treatment commencement.

## Additional file

Additional file 1: **Table S1.** Univariable and multivariable analysis of predictors of MTX non-response for those with active disease at baseline. **Table S2.** Univariable and multivariable analysis of predictors of failure to achieve low disease activity at 6 months. **Figure S1.** Calibration plot for multivariable prediction model for non-response to MTX for those not in remission at baseline. **Figure S2.** Calibration plot for multivariable prediction model for non-response to MTX for those with active disease at baseline. **Figure S3.** Calibration plot for multivariable prediction model for failure to achieve low disease activity. (DOCX 60 kb)

## Abbreviations

ACPA: Anti-citrullinated protein antibody
ACR: American College of Rheumatology
AUC: Area under the curve
bDMARD: Biological disease-modifying antirheumatic drug
BMI: Body mass index
BMQ: Beliefs about Medicines Questionnaire
CRP: C-reactive protein
csDMARD: Conventional synthetic disease-modifying antirheumatic drug
DAS28: Disease Activity Score in 28 joints
DMARD: Disease-modifying antirheumatic drug
EULAR: European League Against Rheumatism
HADS: Hospital Anxiety and Depression Scale
HAQ: Health Assessment Questionnaire
IMD: Index of Multiple Deprivation
IPQ: Illness Perception Questionnaire
LDA: Low disease activity
MTX: Methotrexate
NPV: Negative predictive value
NSAIDs: Non-steroidal anti-inflammatory drugs
PPV: Positive predictive value
RA: Rheumatoid arthritis
RAMS: Rheumatoid Arthritis Medication Study
RF: Rheumatoid factor
SJC: Swollen joint count
TJC: Tender joint count
UP: Undifferentiated polyarthritis
VAS: Visual analogue scale
