# Supplementary material for: Prediction of primary non-response to methotrexate therapy using demographic, clinical and psychosocial variables: results from the UK Rheumatoid Arthritis Medication Study (RAMS)
Source: Arthritis Res Ther. 2018 Jul 13;20:147. doi: 10.1186/s13075-018-1645-5 (PMC6044018; doi:10.1186/s13075-018-1645-5)

**ADDITIONAL FILE 1**

Table S1: Univariable and multivariable analysis of predictors of MTX non-response for those with active disease at baseline

| **Characteristic** | **Univariable** | | **Stepwise multivariable**  **n = 607, AUC = 0.71 (95% CI (0.66, 0.75))** | |
| --- | --- | --- | --- | --- |
|  | **OR (95% CI)** | ***p*** | **OR (95% CI)** | ***p*** |
| *Demographic and lifestyle factors* | | | | |
| Female sex | 1.33 (0.97, 1.83) | 0.08 |  |  |
| Age (years) | 0.99 (0.98, 1.00) | 0.06 |  |  |
| BMI (kg/m^2^) | 1.05 (1.02, 1.07) | <0.01 | **1.05 (1.02, 1.08)** | **<0.01** |
| Smoking: never | ref | ref |  |  |
| Smoking: former | 1.17 (0.84, 1.62) | 0.35 |  |  |
| Smoking: current | 1.65 (1.12, 2.42) | 0.01 |  |  |
| Alcohol consumption: current | 0.94 (0.70, 1.28) | 0.71 |  |  |
| Alcohol consumption: (units/fortnight) | 1.01 (0.99, 1.02) | 0.36 |  |  |
| Coffee/tea consumption: (cups/day) | 1.05 (0.99, 1.11) | 0.10 |  |  |
| IMD score | 1.01 (1.00, 1.02) | 0.05 |  |  |
| *Disease specific factors* | | | | |
| Symptom duration (months) | 1.00 (1.00, 1.01) | <0.01 |  |  |
| RF positive | 0.62 (0.45, 0.86) | <0.01 | **0.57 (0.40, 0.83)** | **<0.01** |
| Satisfied the 1987 ACR criteria | 0.64 (0.43, 0.96) | 0.03 |  |  |
| HAQ score | 1.09 (0.88, 1.33) | 0.44 |  |  |
| Co-morbidities: 0 | ref | ref |  |  |
| Co-morbidities: 1 | 1.48 (1.04, 2.11) | 0.03 |  |  |
| Co-morbidities: 2+ | 1.73 (1.21, 2.47) | <0.01 |  |  |
| Creatinine (mg/dl) | 1.01 (1.00, 1.01) | 0.19 |  |  |
| *Disease Activity* | | | | |
| Morning stiffness (minutes) | 1.00 (1.00, 1.00) | 0.32 |  |  |
| TJC28 | 0.98 (0.96, 1.00) | 0.02 |  |  |
| SJC28 | 0.95 (0.92, 0.97) | <0.01 |  |  |
| CRP (mg/l) | 0.99 (0.98, 1.00) | <0.01 |  |  |
| Patient VAS (mm) | 0.99 (0.98, 1.00) | <0.01 |  |  |
| DAS28-CRP | 0.65 (0.56, 0.75) | <0.01 | **0.49 (0.39, 0.60)** | **<0.01** |
| Pain VAS (mm) | 1.00 (0.99, 1.01) | 0.79 |  |  |
| Fatigue VAS (mm) | 1.01 (1.00, 1.01) | 0.06 |  |  |
| *Medication* | | | | |
| Oral or intramuscular steroids: current/recent | 0.97 (0.73, 1.31) | 0.86 |  |  |
| NSAIDs: current | 1.00 (0.74, 1.36) | 1.00 |  |  |
| csDMARDs: current | 1.44 (1.00, 2.06) | 0.05 |  |  |
| csDMARDs: ever | 1.58 (1.13, 2.20) | 0.01 |  |  |
| MTX starting dose (mg/week) | 0.97 (0.93, 1.02) | 0.24 |  |  |
| *Psychosocial factors* | | | | |
| HADS Anxiety | 1.06 (1.02, 1.09) | <0.01 | **1.11 (1.07, 1.16)** | **<0.01** |
| HADS Depression | 1.02 (0.98, 1.06) | 0.27 |  |  |
| BMQ medication necessity | 1.00 (0.95, 1.04) | 0.84 |  |  |
| BMQ medication concerns | 1.04 (1.00, 1.08) | 0.08 |  |  |
| BMQ necessity-concerns | 0.98 (0.95, 1.01) | 0.20 |  |  |
| IPQ negative illness representation | 1.29 (0.94, 1.77) | 0.12 |  |  |

Table S2: Univariable and multivariable analysis of predictors of failure to achieve low disease activity at 6 months

| **Characteristic** | **Univariable** | | **Stepwise multivariable**  **n = 904, AUC = 0.73 (95% CI (0.70, 0.77))** | |
| --- | --- | --- | --- | --- |
|  | **OR (95% CI)** | ***p*** | **OR (95% CI)** | ***p*** |
| *Demographic and lifestyle factors* | | | | |
| Female sex | 1.71 (1.32, 2.22) | <0.01 |  |  |
| Age (years) | 1.00 (0.99, 1.00) | 0.34 |  |  |
| BMI (kg/m^2^) | 1.07 (1.05, 1.10) | <0.01 | **1.05 (1.02, 1.08)** | **<0.01** |
| Smoking: never | ref | ref |  |  |
| Smoking: former | 0.95 (0.72, 1.24) | 0.69 |  |  |
| Smoking: current | 1.06 (0.77, 1.48) | 0.71 |  |  |
| Alcohol consumption: current | 0.60 (0.46, 0.79) | <0.01 |  |  |
| Alcohol consumption: (units/fortnight) | 0.99 (0.98, 1.00) | 0.03 |  |  |
| Coffee/tea consumptions (cups/day) | 1.02 (0.97, 1.08) | 0.35 |  |  |
| IMD score | 1.02 (1.01, 1.03) | <0.01 |  |  |
| *Disease specific factors* | | | | |
| Symptom duration (months) | 1.00 (1.00, 1.00) | 0.01 |  |  |
| RF positive | 0.69 (0.52, 0.92) | 0.01 |  |  |
| Satisfied the 1987 ACR criteria | 1.07 (0.77, 1.48) | 0.69 |  |  |
| HAQ score | 2.50 (2.07, 3.02) | <0.01 | **1.63 (1.30, 2.05)** | **<0.01** |
| Co-morbidities: 0 | ref | ref |  |  |
| Co-morbidities: 1 | 1.67 (1.25, 2.24) | <0.01 |  |  |
| Co-morbidities: 2+ | 1.90 (1.41, 2.57) | <0.01 |  |  |
| Creatinine (mg/dl) | 0.99 (0.99, 1.00) | 0.15 |  |  |
| *Disease Activity* | | | | |
| Morning stiffness (minutes) | 1.00 (1.00, 1.00) | 0.01 |  |  |
| TJC28 | 1.10 (1.08, 1.12) | <0.01 | **1.06 (1.04, 1.09)** | **<0.01** |
| SJC28 | 1.06 (1.04, 1.09) | <0.01 |  |  |
| CRP (mg/l) | 1.01 (1.00, 1.02) | <0.01 |  |  |
| Patient VAS (mm) | 1.02 (1.01, 1.02) | <0.01 |  |  |
| DAS28 | 1.70 (1.53, 1.89) | <0.01 |  |  |
| Pain VAS (mm) | 1.02 (1.01, 1.02) | <0.01 |  |  |
| Fatigue VAS (mm) | 1.02 (1.02, 1.03) | <0.01 |  |  |
| *Medication* | | | | |
| Oral or intramuscular steroids: current/recent | 1.16 (0.91, 1.49) | 0.24 |  |  |
| NSAIDs: current | 1.07 (0.82, 1.38) | 0.63 |  |  |
| csDMARDs: current | 1.47 (1.07, 2.02) | 0.02 |  |  |
| csDMARDs: ever | 1.51 (1.13, 2.02) | 0.01 |  |  |
| MTX starting dose (mg/week) | 0.96 (0.93, 1.00) | 0.08 |  |  |
| *Psychosocial factors* | | | | |
| HADS Anxiety | 1.14 (1.10, 1.17) | <0.01 | **1.08 (1.04, 1.12)** | **<0.01** |
| HADS Depression | 1.14 (1.10, 1.18) | <0.01 |  |  |
| BMQ medication necessity | 1.06 (1.03, 1.10) | <0.01 |  |  |
| BMQ medication concerns | 1.04 (1.01, 1.08) | 0.02 |  |  |
| BMQ necessity-concerns | 1.01 (0.98, 1.03) | 0.49 |  |  |
| IPQ negative illness representation | 2.34 (1.80, 3.04) | <0.01 |  |  |

Figure S1: Calibration plot for multivariable prediction model for non-response to MTX for those not in remission at baseline


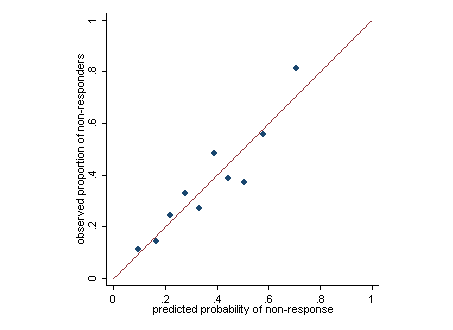


Figure S2: Calibration plot for multivariable prediction model for non-response to MTX for those with active disease at baseline


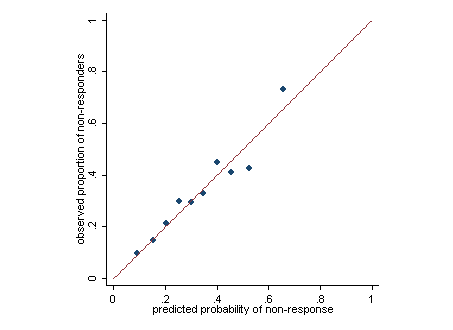


Figure S3: Calibration plot for multivariable prediction model for failure to achieve low disease activity


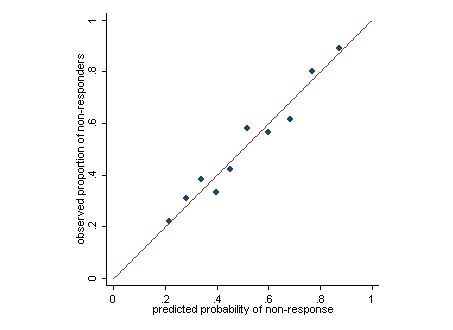

Supplement: Supplementary file 1 — Table S1. Univariable and multivariable analysis of predictors of MTX non-response for those with active disease at baseline. Table S2. Univariable and multivariable analysis of predictors of failure to achieve low disease activity at 6 months. Figure S1. Calibration plot for multivariable prediction model for non-response to MTX for those not in remission at baseline. Figure S2. Calibration plot for multivariable prediction model for non-response to MTX for those with active disease at baseline. Figure S3. Calibration plot for multivariable prediction model for failure to achieve low disease activity. (DOCX 60 kb) [file 13075_2018_1645_MOESM1_ESM.docx]
